# Mr. Stuart Charles Bloxham

**Published:** 1910-09-17

**Authors:** 


### OBITUARY.

The death is announced at Newport, Mon., of Mr. Stuart Charles Bloxham, L.R.C.P., L.R.C.S.Edin., L.F.P.S.Glasg., and L.M., aged fifty-three.

